# Diversity and spillover risk of swine acute diarrhea syndrome and related coronaviruses in China and Southeast Asia

**DOI:** 10.1128/mbio.01197-24

**Published:** 2025-08-19

**Authors:** Alice Latinne, Li-Biao Zhang, Cadhla Firth, Guangjian Zhu, Kevin J. Olival, Cecilia A. Sánchez, Shirley Chen, Noam Ross, Hongying Li, Aleksei A. Chmura, Peter Daszak

**Affiliations:** 1 EcoHealth Alliance 168529, New York, New York, USA; 2 Guangdong Key Laboratory of Animal Conservation and Resource Utilization, Guangdong Public Laboratory of Wild Animal Conservation and Utilization, Institute of Zoology, Guangdong Academy of Sciences 721506 , Guangzhou, China; Washington University in St Louis School of Medicine, St. Louis, USA; University of Chicago, Chicago, Illinois, USA

**Keywords:** alphacoronavirus, bat, Chiroptera, rhinacovirus, zoonoses, SADS

## Abstract

Bats are the reservoir hosts of emerging coronaviruses (CoVs) affecting human and livestock health. We assessed the diversity, evolution, and geographic distribution of two alphacoronaviruses (subgenus *Rhinacovirus*) with considerable potential for emergence: swine acute diarrhea syndrome coronavirus (SADS-CoV), which has caused large outbreaks in pigs in China and can infect primary human airway epithelial cells *in vitro*; and the related *Rhinolophus bat coronavirus HKU2* (HKU2-CoV). Phylogenetic analyses of 523 rhinacovirus sequences from bats in China and Southeast Asia suggest these viruses should be reclassified into at least two distinct CoV species representing two well-supported monophyletic clades. Stronger phylogenetic clustering by sampling location than by host species suggests infrequent long-distance transmission of rhinacoviruses in southern China. Ancestral state reconstruction analysis indicates that *R. sinicus/thomasi* and *R. affinis* have played an important role in rhinacovirus evolution in southern China and that *R. affinis* is the likely reservoir host of SADS-CoV that spilled over into pigs. We used species distribution modeling of *Rhinolophus* spp. bat hosts of rhinacoviruses, combined with pig and human density data, to identify potential geographic rhinacovirus spillover risk in Southeast Asia. Areas of high pig density within suitable bat habitat exist primarily in southern China and northern Vietnam, and hotspots of the highest human density within suitable bat habitat are primarily along the southern coast of China, Java, and central Thailand. Targeted surveillance of pigs and people in these regions may facilitate the timely detection of bat CoV spillover events and mitigate the risk of future outbreaks.

**IMPORTANCE:**

Bats are the reservoir or ancestral hosts of important emerging coronaviruses affecting people (e.g., SARS-CoV and SARS-CoV-2) and livestock (e.g., PEDV, SADS-CoV). Here, we analyzed 523 genetic sequences of SADS-CoV that caused large-scale die-offs of pigs in China, which is known to be able to infect human cells and related HKU2-CoVs. We used this information to identify the horseshoe bat *Rhinolophus affinis* as the likely spillover host for the outbreak in pigs, and identified the bat species within which these viruses evolved. We then modeled the distribution of these host species and their overlap with dense human and pig populations to identify the regions where surveillance programs can help identify spillover events and prevent future outbreaks.

## INTRODUCTION

Coronaviruses (CoVs) are a large group of RNA viruses infecting birds and mammals that are characterized by high zoonotic potential due to their ability to infect a broad range of host species and rapidly adapt to new hosts following cross-species transmission (1–3). Certain bat taxa have been identified as the likely reservoir hosts of the progenitors of several zoonotic CoVs affecting human health, including HCoV-229E, HCoV-NL-63, severe acute respiratory syndrome (SARS)-CoV, Middle East respiratory syndrome (MERS)-CoV, and SARS-CoV-2 that emerged in China and caused a global pandemic (4–8). Rodents are likely ancestral hosts for other human CoVs, including HCoV-HKU1 and HCoV-OC43 (9, 10).

Emerging CoVs also cause important diseases of livestock, including some that have led to considerable economic losses to the pork industry (11, 12). As a major global producer of pigs, Asia is a high-risk area for the emergence of infectious diseases in pig populations. China maintains the highest proportion of the global population of intensively and extensively raised pigs, with most production systems located in an area covering less than one-third of the country, along the Yangtze River and in northern China (13–15). High densities of extensively raised pigs also occur in Vietnam, Thailand, the Philippines, and Myanmar (14). At least six CoVs currently affect pigs worldwide (16). Most recently, a coronavirus-associated fatal swine acute diarrhea syndrome (SADS) emerged in pig farms in China in late 2016 (17–20). The causative agent, SADS-CoV, previously named Porcine Enteric Alphacoronavirus (PEAV) or Swine Enteric Alphacoronavirus (SeACoV), is associated with severe diarrhea with high morbidity and mortality in newborn piglets (18, 20, 21). Clinical signs of SADS-CoV infection are similar to those caused by other swine enteric coronaviruses, such as Porcine Epidemic Diarrhea Virus (PEDV), highlighting the importance of molecular tools for differential diagnosis and viral discovery. SADS-CoV is an HKU2-related CoV, an alphacoronavirus belonging to the subgenus Rhinacovirus, that has been previously identified in horseshoe bats (*Rhinolophus* spp.) in China and Southeast Asia (22–24). Four main phylogenetic groups were identified within rhinacoviruses, suggesting an allopatric evolution of rhinacoviruses on Hainan Island (25). Previous studies suggested that the ancestor of SADS-CoV originated from *Rhinolophus affinis* in Guangdong province, but the direct progenitor of SADS-CoV is still unknown (25, 26). It should also be noted that, while the largest part of the HKU2-CoV and SADS-CoV genomes belongs to alphacoronaviruses, their spike protein genes are more similar to those of betacoronaviruses, and it has been suggested that these viruses emerged from a recombination event between an alphacoronavirus and a betacoronavirus spike protein gene (23, 27, 28).

A retrospective study identified SADS-CoV in pig farms in Guangdong province from August 2016, with evidence of later spread to several farms in Guangdong and Fujian provinces, causing major economic losses (20, 29, 30). Since then, SADS-CoV was identified in diarrheal disease outbreaks in an intensive pig farm in Guangxi province that killed more than 3,000 piglets in May 2021 and in a pig farm in Henan province in June 2023, with phylogenetic and recombination analysis of the genomes from both outbreaks suggesting that SADS-CoV has continued to circulate in pigs in China since its first known spillover and outbreak in 2016 (31, 32).

Recent work has demonstrated that SADS-CoV can replicate efficiently in primary human lung and intestinal cells *in vitro*, showing clear potential for human infection (33–35). In addition, SADS-CoV causes a lethal infection in suckling mice that includes a neuroinflammatory response (36). Conditions for human infection with SADS-CoV may occur where people and bats have high contact (e.g., occupational exposure in caves, or exposure of people who live near bat roosts or foraging sites) or where people and pigs have contact, particularly during SADS-CoV outbreaks when extensive virus replication and recombination may occur. While SADS-CoV has only thus far been detected in pig populations in China, the purported wildlife reservoir hosts of this group of coronaviruses, *Rhinolophus* spp. bats, are widely distributed across Southeast Asia (6). Thus, the spillover risk of SADS-CoV and other HKU2-related CoVs (hereafter SADS-related coronaviruses, SADSr-CoVs) is not limited to China, nor only to pig populations. Hotspots of rhinolophid species diversity occur in tropical forest ecosystems throughout mainland Southeast Asia, with the highest diversity observed in large contiguous areas encompassing southern China, Laos, and Vietnam, with smaller hotspots in northern Myanmar, eastern Thailand, and Cambodia (6). In this study, we aimed to assess the diversity of SADSr-CoVs in China and Southeast Asia and identify the key natural reservoir species involved in the circulation and initial spillover of SADS-CoV into pigs using an unprecedented data set of more than 500 sequences. Using bat host distribution modeling and pig and human population data, we also aimed to identify geographic areas with the greatest overlap between bats, pigs, and humans in China and Southeast Asia. This information can be used to target surveillance efforts to help mitigate the risk of future SADS-CoV and related virus spillover.

## MATERIALS AND METHODS

### Bat sampling

Bats were captured at roost sites or feeding areas using mist nets and harp traps and placed individually in clean cotton bags prior to sampling. Bats were identified to species using external morphology by trained field scientists. Oral and rectal swabs were collected from individual bats using manual restraint. Fresh fecal pellets were collected from tarps placed below bat colonies. Duplicate samples were collected in either TRIzol (Invitrogen) lysis buffer or viral transport medium and moved to liquid nitrogen directly before storing at −80°C until needed. A wing punch was collected for molecular host species confirmation (i.e., DNA barcoding) purposes and stored in ethanol. All sampling was non-lethal, and bats were released at the site of capture immediately after sample collection. All personnel involved in bat capture and handling wore personal protective equipment (i.e., N95 respirator, eye protection, double nitrile gloves, and dedicated clothing) during bat trapping and sampling. Bat handling and biosafety methods were approved via an interinstitutional agreement with EcoHealth Alliance under the Tufts University IACUC (Protocol #G2017-32), UC Davis IACUC (Protocol #19300), and locally approved via the Wuhan Institute of Virology Chinese Academy of Sciences IACUC (Protocol WIVA05201705) and Animal Ethics Committee of Institute of Zoology, Guangdong Academy of Sciences (Approval No. GIABR20200810).

### PCR screening

RNA was extracted from 200 µL of lysis buffer containing rectal swab samples or fecal pellets with the High Pure Viral RNA Kit (Roche) following the manufacturer’s instructions. RNA was eluted in 50 µL of elution buffer before storage at −80°C. A hemi-nested RT-PCR (Invitrogen) was used to detect CoV RNA using a set of primers targeting a 440-nt fragment of the RNA-dependent RNA polymerase (RdRp) gene and optimized for bat CoV detection (CoV-FWD3: GGTTGGGAYTAYCCHAARTGTGA; CoV-RVS3: CCATCATCASWYRAATCATCATA; CoV-FWD4/Bat: GAYTAYCCHAARTGTGAYAGAGC) (37). First-round PCR was performed as follows: 50°C for 30 min, 94°C for 2 min, followed by 40 cycles consisting of 94°C for 20 s, 50°C for 30 s, 68°C for 30 s, and a final extension step at 68°C for 5 min. Second-round PCR was performed as follows: 94°C for 2 min followed by 40 cycles consisting of 94°C for 20 s, 59°C for 30 s, 72°C for 30 s, and a final extension step at 72°C for 7 min. PCR products were gel-purified and sequenced with an ABI Prism 3730 DNA analyzer (Applied Biosystems, USA). PCR products with low concentration or that yielded poor-quality sequences were cloned into pGEM-T Easy Vector (Promega) for sequencing. Host DNA barcoding was attempted on all 186 samples to confirm field species identification using PCR protocols targeting the mitochondrial cytochrome b (CYTB) or NADH dehydrogenase subunit 1 (ND1) genes (38). Unambiguous host species identification was possible for a high proportion (87%) of these samples (162/186).

### Sequence data

Nucleotide sequences were aligned using MUSCLE (39) and the alignment refined manually in Geneious 9.1.8. (https://www.geneious.com). Our final data set included 186 *Rhinacovirus* RdRp sequences generated for this study from Chinese bats. The GenBank accession numbers for all sequences generated for this study can be found in Supplemental Table 1 (available at https://osf.io/rcepg/?view_only=f673c894413a44df83716db155aad489 and https://naturehealthglobal.org/latinne-et-al-2025-supplemental/). We also added 337 sequences from GenBank to the data set, including 42 SADS-CoV sequences from pigs in Chinese farms available in GenBank (see Supplemental Table 1 at https://osf.io/rcepg/?view_only=f673c894413a44df83716db155aad489 and https://naturehealthglobal.org/latinne-et-al-2025-supplemental/). We selected GenBank sequences for which information about the sampling location (at least to a province-level) was available. The host of 503 of these sequences was identified as a species. We were able to confirm host species identifications using a barcoding sequence for 352 of these *Rhinacovirus* sequences for which a CYTB or ND1 sequence was available from this study and others.

The host species *R. sinicus* and *R. thomasi* were grouped in our data set (“*R. sinicus/thomasi*”) as they belong to a species complex with frequent hybridization and introgression events and among which taxonomic boundaries are not fully resolved (40). The host species of samples characterized by CYTB barcoding sequences highly similar to sequences identified as *Rhinolophus* sp. 2 (99.8%) (41) and *Rhinolophus stheno* (98.7%) (42, 43) was named “*Rhinolophus sp. 2/R. stheno*” in our data set.

### Phylogenetic analyses

Bayesian phylogenetic trees of the RdRp sequences were constructed using MrBayes v3.2.7 (44) and a GTR+Γ4 + I model of nucleotide substitution. Three phylogenies were estimated: one containing the complete data set (*n* = 523), a second containing only sequences from a known host (known host data set; *n* = 503), and a third one including only sequences from bat hosts identified using a DNA barcoding sequence and those from swine hosts (barcoded data set; *n* = 352). For each phylogeny, two independent runs were performed in MrBayes, with sampling every 10,000 generations, and terminated when the standard deviation of split frequencies reached <0.01. The posterior distribution of trees, Bayesian posterior probabilities (BPP), and model parameters were summarized from the MCMC sampling, and a maximum clade credibility (MCC) tree was created by summarizing the trees from each run after the initial 10% of trees were discarded as burn-in. Each phylogeny was initially rooted using a representative γ-CoV (Avian infectious bronchitis virus, GenBank Accession numbers NC_001451.1 and KX398025.1) and β-CoV (HKU9, GenBank Accession numbers HM211101.1 and HM211099.1) as outgroups. A median-joining network (45) was reconstructed using the complete and known host data sets and the software PopArt 1.7 (46). We also reconstructed the Maximum Likelihood phylogeny of the Spike gene (S gene) using PhyML 3.0 (47) and amino acid S gene sequences from 54 of the 523 samples for which a whole-genome sequence was available in GenBank.

To assess the extent of spatial and host structure present in the two phylogenies, we used the program BaTS (Bayesian Tip-association Significance testing) (48). This method accounts for phylogenetic uncertainty in investigating phylogeny-trait correlations, with traits assigned to each sequence based on either the location of sampling (province) or host. We used the 95% credible set of trees from each Bayesian posterior distribution and performed 1,000 random permutations of tip states to estimate a null distribution for each statistic. BaTS outputs an Association Index (AI) and a Parsimony Score (PS) for each input phylogeny, whereby computing the index ratios of the observed to expected distributions allows an assessment of the strength of the association between each location/host and the associated phylogeny. An index ratio of zero is suggestive of population subdivision in relation to the trait being analyzed, and a value of one is indicative of random mixing (i.e., panmixia). BaTS also returns a value for the maximum Monophyletic Clade (MC) size for each character state that allows an assessment of the level of phylogenetic clustering by individual locations/hosts across the whole phylogenetic tree relative to the null (expected) distribution.

We also calculated the mean phylogenetic distance (MPD) and its standardized effect size (SES) (49) among rhinacovirus sequences for each host and province using the R package picante (50) and the MCC trees from MrBayes. MPD measures the mean phylogenetic distance among all pairs of sequences within a host or province. It reflects phylogenetic structuring across the whole phylogenetic tree and assesses the overall divergence of CoV sequences observed in a community. SES MPD values correspond to the difference between the phylogenetic distances in the observed communities versus null communities. Low and negative SES values denote phylogenetic clustering, high and positive values indicate phylogenetic overdispersion, while values close to 0 indicate random dispersion. The SES values were calculated by building null communities by randomly reshuffling tip labels 1,000 times across the entire phylogeny.

Finally, to reconstruct the ancestral states for both location and host at each node in the phylogeny based on the complete and known host data set, respectively, we used the MultiState model of discrete trait evolution in the program BayesTraits and the maximum likelihood method of model-fitting (51). To confirm the absence of bias due to potential misidentification of bat species, we also performed ancestral state reconstructions for hosts using the barcoded data set. As input, we used the MCC trees generated by MrBayes with branch lengths and reported values only for those nodes with BPP ≥65. As our data set was heavily biased towards sampling from only a few hosts (e.g., sequences generated from *R. affinis* samples comprised 46% of all sequences from a barcoded host), we randomly subsampled our barcoded data set 10 times to include a maximum of 15 sequences from each host/location (*n* = 142). We then inferred Bayesian phylogenetic trees for each of these 10 random subsets as above and performed ancestral state reconstructions on the resulting subset phylogenies using BayesTraits.

### Bat host distribution modeling

For host distribution modeling, we focused on four *Rhinolophus* species that were most commonly detected as rhinacovirus hosts: *R. affinis*, *R. pusillus*, *R. sinicus*/*thomasi,* and *R. stheno*. We used species distribution modeling to spatially delineate suitable habitat for these species and then intersected these maps with pig and human density data in China and countries within Southeast Asia. We analyzed a broader geographic extent than is used for political definitions of Southeast Asia to include parts of South Asia, due to the extensive range of some of the bat species and in line with our previous work (52). All modeling was performed in the R statistical environment v 4.2.1 (53).

We obtained recent (within the last 50 years) species occurrence points from several sources. We sourced occurrences from the Global Biodiversity Information Facility (GBIF) database (54) using the rgbif package (55, 56). We also sourced data from DarkCideS 1.0 v4 (57), the USAID PREDICT project (58), and multiple literature sources (59–65). We removed duplicate records, geographic outliers, records with inaccurate or imprecise coordinate information, records located near country and province capitals or centroids, and records located near GBIF headquarters or biodiversity institutions using the CoordinateCleaner package (66). We also removed records that were missing data for our final set of predictor variables (see below). These cleaning steps resulted in 414 unique occurrence points for *R. affinis*, 224 for *R. pusillus*, 214 for *R. sinicus/thomasi*, and 55 for *R. stheno*. To delimit the study area for each species, we set a 250–300 km buffer around its occurrence points using the terra package (67) and excluded countries that appeared insufficiently surveyed.

We considered a number of climatic and landscape variables that might influence species occurrence, including 19 bioclimatic variables (68), elevation (68), percent land cover (17 classes) (69), carbonate rocks (used as a proxy for karst landscape and cave distribution—hereafter, karst) (70), human population density (71), and median nighttime light intensity (72). Land cover, human population density, and nighttime light data were available for the year 2020; karst data were published in 2017, and bioclimatic variables were available as averages for the years 1970–2000. From the land cover classes, we selected six that we considered likely to be relevant to our study species (percent water bodies, percent evergreen needleleaf forests, percent evergreen broadleaf forests, percent deciduous needleleaf forests, percent deciduous broadleaf forests, and percent mixed forests). We resampled human population density, nighttime light data, bioclimatic variables, and elevation data to the resolution of the land cover data (0.05°, ~5.6 km at the equator) using bilinear interpolation via the terra package. We rasterized a shapefile of karst distribution, then calculated distance to karst using the whitebox package (73, 74). We tested for collinearity among the set of 29 predictor variables using the ENMTools package (75). When a pair of predictor variables had a Pearson correlation coefficient exceeding an absolute value of 0.7, we retained only one variable, prioritizing variables with more relevant biological meaning. Finally, we visually inspected all remaining predictor layers and removed percent deciduous needleleaf forests, as it was nearly uniform across the study range. This process retained a final set of 11 predictor variables: annual mean temperature (bio1), annual precipitation (bio12), precipitation seasonality (bio15), nighttime light intensity, human population density, distance to karst, percent water bodies, percent evergreen needleleaf forests, percent evergreen broadleaf forests, percent deciduous broadleaf forests, and percent mixed forests. For each species, we clipped these 11 predictor variables to the corresponding study area described above.

To reduce potential effects of sampling bias, we performed spatial thinning on occurrence points using the fuzzySim package (76) to match the resolution of the predictor variables. This reduced the number of occurrence points to 270 for *R. affinis*, 141 for *R. pusillus*, 161 for *R. sinicus/thomasi*, and 33 for *R. stheno*. Due to the small number of occurrence points for *R. stheno*, we did not pursue further modeling for this species. For the remaining species, unoccupied cells within the study area were chosen as background points, then randomly subsampled using the fuzzySim package to produce an occurrence:background ratio of 1:100. Using the blockCV package (77), we divided the study area for each species into a hexagonal grid, then randomly assigned the hexagons into five folds, with roughly equal numbers of occurrence and background points in each fold.

We modeled *Rhinolophus* species distributions using maximum entropy (Maxent) (78) implemented via the ENMeval package (79, 80). We performed model tuning by assessing four feature classes (linear, linear-quadratic, hinge, and linear-quadratic-hinge) and nine regularization multipliers (1–5 by increments of 0.5) for a total of 36 candidate models per species. We implemented cross-validation using the folds described above, where one fold, in turn, was reserved for model evaluation and the other folds were used for model training. We used the cross-validation results to select the optimal model for each species by choosing the candidate model with the lowest average omission rate (OR10); in case of ties, the model with the highest validation AUC (area under the receiver operator curve) was chosen (81, 82). We assessed the contribution of predictor variables to the model using permutation importance, where larger percentage values indicate greater importance (83). We projected the optimal model for each species on its geographic range as defined by the IUCN Red List (84), combining the individual IUCN ranges for *R. sinicus* and *R. thomasi* into a single range. Red List assessments for the hosts were last performed in 2018–2019. Raw model output was transformed using the complementary log-log (cloglog) method to produce a continuous estimate of habitat suitability from 0 to 1 (see Supplemental Fig. 1. at https://osf.io/rcepg/?view_only=f673c894413a44df83716db155aad489 and https://naturehealthglobal.org/latinne-et-al-2025-supplemental/). We then converted this continuous output to a binary map of suitable/unsuitable habitat by applying a tenth percentile training presence threshold (81). We combined individual species maps to create a single map displaying suitable habitat for at least one host species.

We intersected the bat habitat map with a pig density raster to identify regions of high pig density within suitable habitat for rhinacovirus bat hosts, as high density might allow for onward pig-to-pig transmission following an initial spillover event. In the absence of readily available and consistent data on pig farms at a subnational level, we used global pig distribution data (dasymetric product) from the Gridded Livestock of the World, version 4.0 (85, 86). The mean pig density within the spatial extent encompassing suitable bat host habitat was ~42 pigs/km^2^; therefore, we set a lower bound of >40 pigs/km^2^ for visualization purposes. We also calculated and visualized the number of pigs at risk of SADSr-CoV spillover by Chinese province, defining “at risk” as living within a suitable bat host habitat area. Given recent findings demonstrating that SADS-CoV can replicate efficiently in human lung and intestinal cells (33), we similarly intersected the bat distribution map with a human population density raster (71) to visualize areas of high human density within suitable habitat for rhinacovirus bat hosts. We set a lower bound of >275 people/km^2^ for visualization, based on a mean population density value of ~281 people/km^2^ within the spatial extent encompassing suitable host habitat. We calculated the number of humans at risk of spillover within Chinese provinces as above. Though many factors are involved in the process of spillover (87), overlap between reservoir hosts and recipient hosts is likely an important prerequisite for most spillover events, and identifying these areas of overlap can guide targeted surveillance efforts (52).

## RESULTS

### Phylogenetic analyses of *Rhinacovirus* sequences

Samples from a total of 15 bat species and four genera (*Rhinolophus*, *Hipposideros*, *Myotis*, and *Tylonycteris*) harbored *Rhinacovirus* sequences (Table 1). Most of these sequences were detected in *R. affinis* (*n* = 232), *R. sinicus/thomasi* (*n* = 85), *R. sp. 2/R*. *stheno* (*n* = 60), and *R. pusillus* (*n* = 64) (Table 1). Rhinacoviruses were primarily identified in samples from the Southern Chinese provinces of Guangdong, Yunnan, and Guangxi, but these viruses have also been detected in Thailand (Table 2). No rhinacovirus sequences have been reported in samples from Northern China. It was not possible to derive prevalence of infection estimates (% of samples positive by PCR) due to a lack of data on overall sample size (i.e., lack of negative data) for most of the GenBank data.

**TABLE 1:** Number of *Rhinacovirus* RdRp sequences available for each host species, observed MPD, and its SES within each host^*a*^

Host species	*n*	MPD observed	MPD random	MPD random SD	MPD observed rank	SES MPD	*P*-value
*Rhinolophus affinis*	232	0.047	0.070	0.002	1.000	−11.800	**0.001**
*Rhinolophus sinicus/* *thomasi*	85	0.051	0.070	0.004	1.000	−4.758	**0.001**
*Rhinolophus pusillus*	64	0.038	0.070	0.005	1.000	−6.911	**0.001**
*Rhinolophus sp. 2/R. stheno*	60	0.016	0.070	0.005	1.000	−10.785	**0.001**
*Rhinolophus rex*	7	0.004	0.070	0.016	1.000	−4.034	**0.001**
*Rhinolophus malayanus*	3	0.102	0.069	0.027	824.000	1.232	0.823
*Rhinolophus* *ferrumequinum*	2	0.147	0.071	0.038	970.000	2.003	0.969
*Rhinolophus shameli*	1	NA^*b*^	NA	NA	NA	NA	NA
*Rhinolophus macrotis*	1	NA	NA	NA	NA	NA	NA
*Rhinolophus siamensis*	1	NA	NA	NA	NA	NA	NA
*Rhinolophus pearsonii*	1	NA	NA	NA	NA	NA	NA
*Hipposideros armiger*	1	NA	NA	NA	NA	NA	NA
*Myotis laniger*	1	NA	NA	NA	NA	NA	NA
*Myotis ricketti*	1	NA	NA	NA	NA	NA	NA
*Tylonycteris robustula*	1	NA	NA	NA	NA	NA	NA
*Sus scrofa* (swine)	42	0.006	0.070	0.006	1.000	−10.608	**0.001**
Unknown bat host	20	NA	NA	NA	NA	NA	NA

^*a*^ One-tailed *P*-values (quantiles) were calculated after randomly reshuffling tip labels 1,000 times along the entire phylogeny (MPD random). Significant *P*-values are highlighted in bold (α = 0.05).

^*b*^ NA means not applicable.

**TABLE 2:** Number of *Rhinacovirus* RdRp sequences available for each province, observed MPD, and its SES within each province^*a*^

Province	*n*	MPD observed	MPD random	MPD random SD	MPD observed rank	MPD SES	*P*-value
Yunnan	218	0.045	0.070	0.002	1.000	−12.549	**0.001**
Guangdong	184	0.052	0.070	0.002	1.000	−7.712	**0.001**
Guangxi Zhuang AR	50	0.048	0.070	0.005	1.000	−4.163	**0.001**
Hainan	12	0.036	0.071	0.012	1.000	−2.934	**0.001**
Zhejiang	11	0.053	0.071	0.012	88.000	−1.468	0.088
Fujian	16	0.071	0.070	0.010	513.000	0.081	0.512
Jiangxi	16	0.015	0.071	0.010	1.000	−5.717	**0.001**
Hong Kong SAR	6	0.004	0.070	0.017	1.000	−3.811	**0.001**
Hubei	4	0.010	0.071	0.022	1.000	−2.746	**0.001**
Hunan	3	0.014	0.071	0.027	10.000	−2.116	**0.010**
Thailand	1	NA^*b*^	NA	NA	NA	NA	NA
Anhui	1	NA	NA	NA	NA	NA	NA
Guizhou	1	NA	NA	NA	NA	NA	NA

^*a*^ One-tailed *P*-values (quantiles) were calculated after randomly reshuffling tip labels 1,000 times along the entire phylogeny (MPD random). Significant *P*-values are highlighted in bold (α = 0.05).

^*b*^ NA means not applicable.

**Fig 1:**
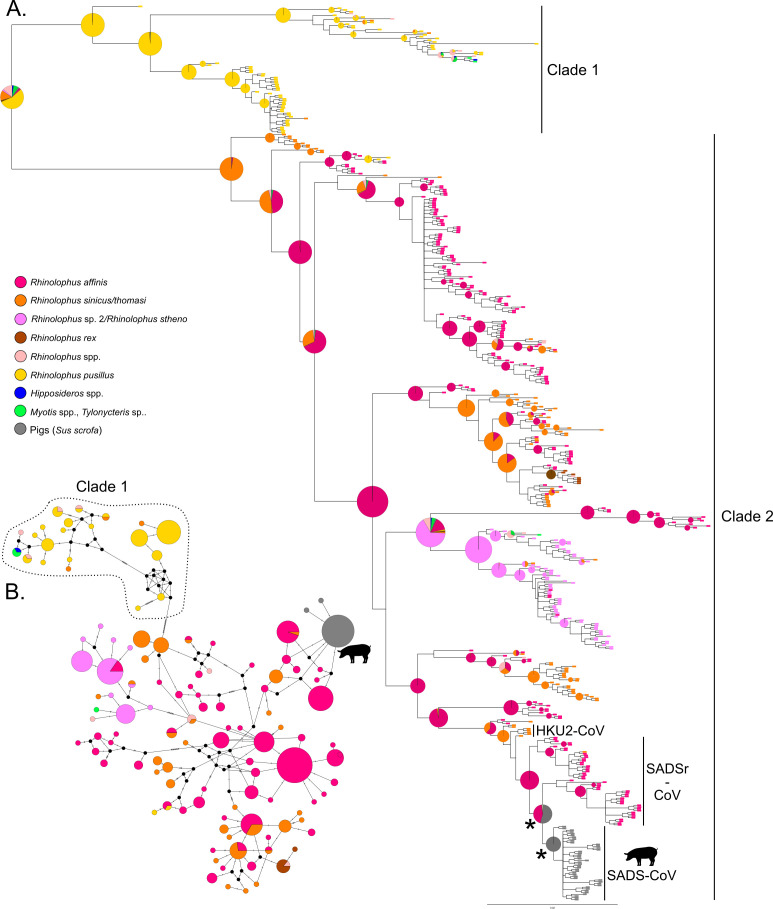
(**A**) Bayesian MCC phylogeny of rhinacoviruses for RdRp sequences with known hosts only. The tip colors indicate the host species associated with each sequence, while the pie graphs show the most likely ancestral host(s) for each clade with BPP ≥65. Nodes with a * are not supported (BPP <65) but are of interest. (**B**) Median-joining network of rhinacoviruses for the data set with known hosts only. Colored circles correspond to distinct rhinacovirus sequences, and circle size is proportional to the number of identical sequences in the data set. Small black circles represent median vectors (ancestral or unsampled intermediate sequences). The numbers of mutational steps between sequences are represented as hatch marks along branches. Circles are colored according to the host species. *Rhinolophus* spp. includes *R. ferrumequinum, R. macrotis, R. malayanus, R. pearsonii, R. shameli,* and *R. siamensis*.

**Fig 2:**
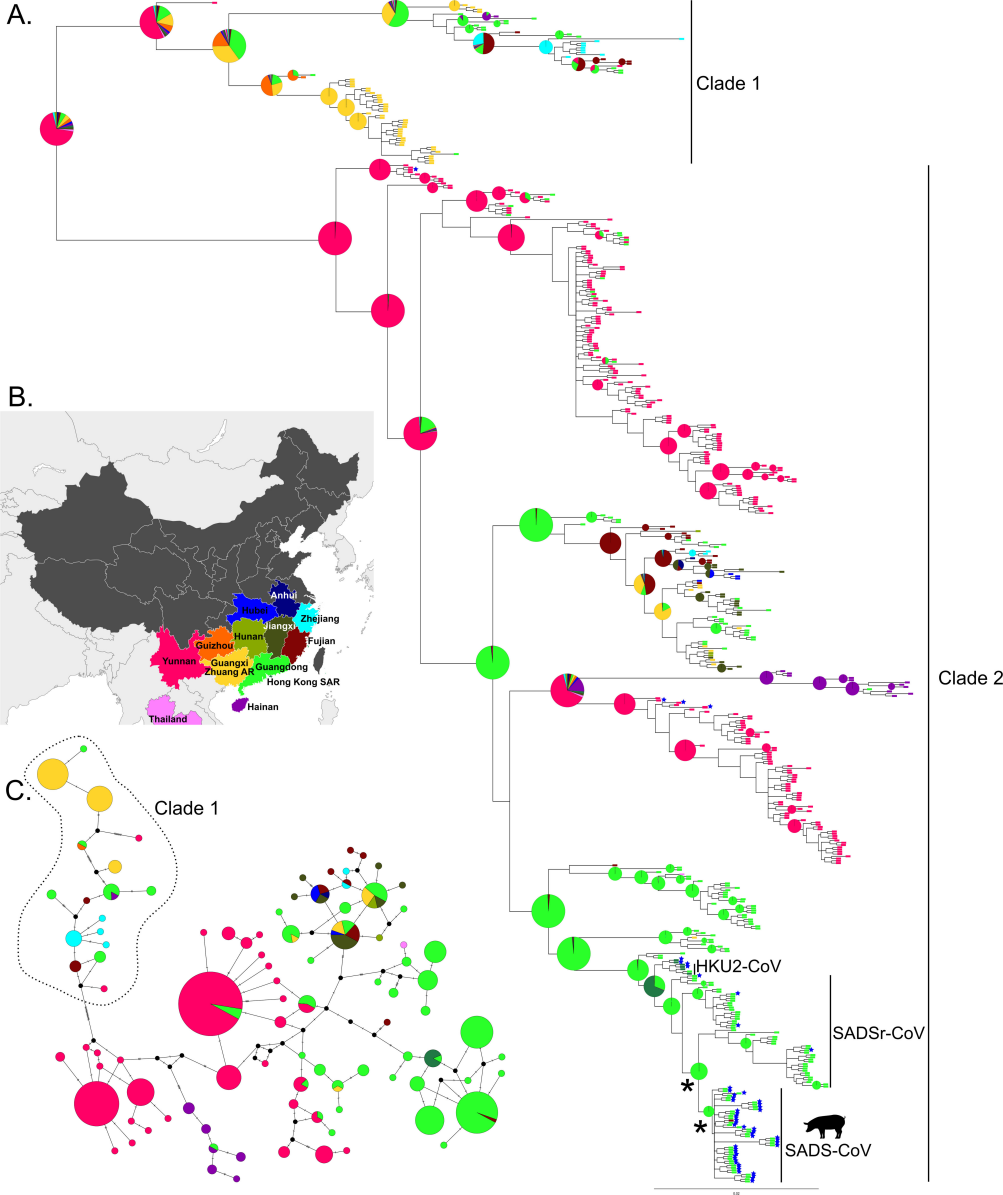
(**A**) Bayesian MCC phylogeny of rhinacoviruses for all RdRp sequences. The tip colors indicate the location (province) of sampling for each sequence, while the pie graphs show the most likely ancestral location(s) for each clade with BPP ≥65. Blue stars show samples for which a whole genome sequence is available in GenBank. Nodes with a * are not supported (BPP <65) but are of interest. (**B**) Map of China and neighboring regions showing the locations where *Rhinacovirus* sequences have been detected. (**C**) Median-joining network of *Rhinacovirus* sequences. Colored circles correspond to distinct *Rhinacovirus* sequences, and circle size is proportional to the number of identical sequences in the data set. Small black circles represent median vectors (ancestral or unsampled intermediate sequences). The numbers of mutational steps between sequences are represented as hatch marks along branches. Circles are colored according to the location of sampling.

Examination of the Bayesian MCC tree obtained from the complete and known host RdRp data sets revealed the presence of two monophyletic clades (BPP = 88 and 92, respectively) within the rhinacoviruses, which were also visible in the median joining networks (Fig. 1 and 2). The average percent nucleotide identity between sequences in Clade 1 vs. Clade 2 was 83.6% (SD = 2.9%). Clade 1 was dominated by sequences derived from *R. pusillu*s (probability of an *R. pusillus* ancestor = 0.99) sampled in Guangxi Zhuang Autonomous Region (AR) and Guangdong Province (Fig. 1 and 2). Clade 2 contained all other rhinacovirus sequences in the data set, including SADS-CoV from pigs and related sequences from bat hosts (SADSr-CoVs) and HKU2-CoV. Clade 2 most likely originated in *R. sinicus/thomasi* (probability of an *R. sinicus/thomasi* ancestor = 0.97; Fig. 1) from Yunnan province (probability of a Yunnan origin = 0.99; Fig. 2). These results were also returned from the analysis of all 10 randomly subsampled data sets, with probabilities ranging from 0.87 to 0.99 for an *R. sinicus/thomasi* ancestor and from 0.95 to 0.99 for a Yunnan origin of Clade 2. The two main clades were also observed in the Bayesian MCC tree obtained from the barcoded data set, which confirmed the origin of Clades 1 and 2 in *R. pusillus* (probability = 0.98) and *R. sinicus/thomasi* (probability = 0.97), respectively (see Supplemental Fig. 2 at https://osf.io/rcepg/?view_only=f673c894413a44df83716db155aad489 and https://naturehealthglobal.org/latinne-et-al-2025-supplemental/).

We found evidence for significant structuring of rhinacoviruses by both geography and host species using trait association tests (AI and PS) (Table 3), rejecting the null hypothesis of no association between sampling location/host and phylogeny in each case. The use of index ratios (i.e., ratio of observed to expected values for AI and PS) allows for the strength of these associations to be characterized further and reveals an overall slightly stronger clustering by location (province) of sampling than by host species (Table 3). However, the AI and PS index ratios for both location and host tended toward zero, which is suggestive of infrequent transmission of rhinacoviruses between hosts and locations in southern China. Examination of the MC size for each location also supported the presence of strong phylogeographic structure in this data set, with significantly more clustering for nine of 13 provinces than expected by chance (*P* ≤ 0.002, Table 3). Of all locations sampled in this study, only those with *N* = 1 (Anhui province, Guangxi Zhuang AR, and Thailand) and *N* = 3 (Hunan Province) sequences did not exhibit strong evidence of phylogeographic clustering (although clustering is not possible for *N* = 1). We also found negative values of the SES MPD, which measures the difference between the mean phylogenetic distance among all pairs of sequences in the observed communities (i.e., within a host or province) versus null communities. These negative and mostly significant values indicated significant phylogenetic clustering of *Rhinacovirus* lineages sampled from bat communities at the same location (Table 2). Only Fujian and Zhejiang provinces were characterized by a non-significant value of SES MPD, indicating random phylogenetic dispersion of sequences. Sequences sampled from Yunnan, Guangdong, and Guangxi provinces formed large, well-supported monophyletic subclades within Clade 2 and were characterized by high negative values of SES MPD (Table 2, Fig. 2).

**TABLE 3:** Phylogeny-trait association tests for sampling location and host for rhinacoviruses^*a*^

Statistic	Cluster	Index ratio, observed to expected (95% CI)	Observed value (95% CI)	Expected value (95% CI)	*P*-value
Association index (AI)					
Location		0.163 (0.132–0.193)	5.47 (4.26–6.72)	33.55 (32.26–34.72)	<<0.001
Host		0.173 (0.146–0.0.98)	5.91 (4.83–6.98)	34.09 (32.97–35.19)	<<0.001
Parsimony score (PS)					
Location		0.233 (0.225–0.242)	58.57 (55.00–62.00)	250.91 (244.46–256.73)	<<0.001
Host		0.202 (0.194–0.208)	50.72 (48.00–53.00)	251.38 (247.18–255.64)	<<0.001
Maximum clade scores (MC)^*b*^					
Location	FJ	NA^*c*^	3 (3–3)	1 (1–1)	0.001
	GD	NA	40 (25–65)	4 (3–4)	0.001
	GX	NA	12 (5–21)	2 (2–2)	0.001
	HB	NA	2 (1–3)	1 (1–1)	0.001
	HI	NA	4 (2–6)	1 (1–1)	0.001
	HK	NA	3 (1–5)	1 (1–1)	0.002
	JX	NA	3 (2–5)	1 (1–1)	0.001
	YN	NA	74 (74–74)	4 (4–5)	0.001
	ZJ	NA	5 (2–9)	1 (1–1)	0.001
Host	*R. affinis*	NA	38 (25–68)	5 (4–6)	0.001
	*R. pusillus*	NA	12 (6–21)	2 (2–2)	0.001
	*R. sinicus/thomasi*	NA	22 (22–22)	2 (2-3)	0.001
	*R. malayanus*	NA	2 (2–2)	1 (1–1)	0.001
	*R. sp. 2/R. stheno*	NA	21 (18–29)	2 (2–2)	0.001
	*R. rex*	NA	4 (2–7)	1 (1–1)	0.001
	*S. scrofa*	NA	18 (7–37)	2 (1-2)	0.001

^*a*^ Lower index ratios suggest higher levels of population structure.

^*b*^ Significant MC scores shown only.

^*c*^ NA means not applicable.

Although the phylogeny-host association analysis supported the presence of phylogenetic clustering by host species, there was less structure by host than by location in this data set (Table 3, Fig. 1). Indeed, of the 16 host species that were included in this analysis, only seven were more clustered than expected across the phylogeny, as indicated by their significant MC scores (Table 3). Notably, sequences derived from *R. affinis*, *R. sp. 2/R. stheno*, and *R. sinicus/thomasi* formed the largest monophyletic clades (Table 3). Estimates of phylogenetic diversity by host (SES MPD) further supported the presence of significant phylogenetic clustering for sequences derived from six of the 16 host species included in our data set, with the highest values observed for *R. affinis*, followed by *R. sp. 2/R*. *stheno*, *R. pusillus,* and *R. sinicus/thomasi* (Table 1). However, while lineages from *R. affinis* and *R. sinicus*/*thomasi* were widely distributed across the phylogeny, those from *R. pusillus* were primarily found within Clade 1 (Table 3, Fig. 1). The bat species *R. affinis* and *R. sinicus/thomasi* were also estimated as the most likely ancestor for the majority of supported nodes in the phylogeny (Fig. 1), further strengthening the suggestion that these two species were the ancestors of several rhinacovirus lineages in southern China. Together, these data suggest that *R. affinis* and *R. sinicus/thomasi* may be important reservoirs of rhinacovirus diversity in southern China.

### Origin of SADS-CoV

All SADS-CoV sequences from pigs formed a single monophyletic clade in the Bayesian MCC RdRp tree within a larger, well-supported clade (SADSr-CoV) dominated by sequences from *R. affinis* (Fig. 1), with an average of 99.2% nucleotide identity between SADS-CoV and SADSr-CoV RdRp sequences. Our ancestral state reconstruction analysis based on the known host RdRp data set and barcoded RdRp data set both strongly supported *R. affinis* as the most likely ancestral host of SADSr- and SADS-CoV in pigs (probability >0.99 in both trees), and this result was identical in all 10 of our randomly subsampled data sets (probability >0.99). The S gene tree also showed the close association between *R. affinis* and pig S gene sequences, with a well-supported clade including all pig and three *R. affinis* sequences (Supplemental Fig. 3 at https://osf.io/rcepg/?view_only=f673c894413a44df83716db155aad489 and https://naturehealthglobal.org/latinne-et-al-2025-supplemental/).

We also found evidence to support the suggestion that the initial spillover event of SADSr-CoV from bats to pigs occurred in Guangdong Province (probability >0.99). This result was also returned in the analysis of all ten randomly subsampled RdRp data sets, with probabilities ≥ 0.99 in all cases. However, most SADS-CoV RdRp sequences were sampled from Guangdong Province within a few months of each other in 2017 (Fig. 1 and Supplemental Table 1 at https://osf.io/rcepg/?view_only=f673c894413a44df83716db155aad489 and https://naturehealthglobal.org/latinne-et-al-2025-supplemental/). The sole SADS-CoV RdRp sequence from Fujian province was identical to those from Guangdong province, which is suggestive of a single spillover event into pigs followed by ongoing pig transmission (Fig. 2). The only available SADS-CoV sequence from 2019 was also identical to those from 2017 in the RdRp region used in this study.

### Host distribution modeling

Ecological and bioclimatic predictor variables varied for each rhinacovirus host species distribution model (as assessed by permutation importance), but percent broadleaf forest cover and annual mean temperature were consistently important variables across species. For *R. affinis*, percent evergreen broadleaf forests (37.4%), percent deciduous broadleaf forests (25.0%), annual mean temperature (bio1; 21.4%), precipitation seasonality (bio15; 6.1%), and distance to karst (5.6%) were the most important predictors (i.e. >5% permutation importance). For *R. pusillus*, the most important predictors were percent deciduous broadleaf forests (43.0%), percent evergreen broadleaf forests (17.1%), annual mean temperature (15.1%), distance to karst (14.5%), and nighttime light intensity (7.3%). For *R. sinicus/thomasi*, the most important predictors were annual precipitation (bio12; 42.1%), annual mean temperature (20.4%), distance to karst (18.5%), percent evergreen broadleaf forests (7.4%), and human population density (6.4%).

**Fig 3:**
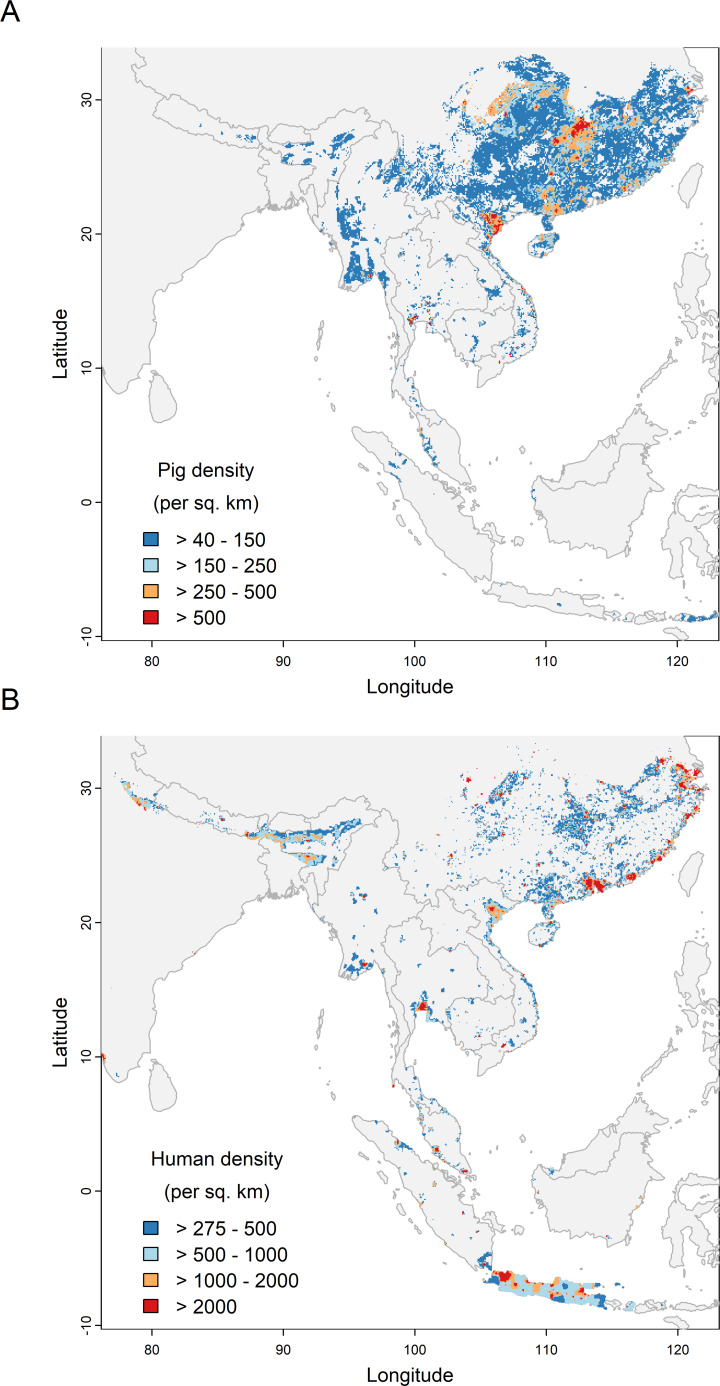
Areas of predicted suitable habitat for one or more *Rhinolophus* spp. rhinacovirus reservoir hosts (*R. affinis*, *R. pusillus*, and *R. sinicus/thomasi*), intersected with (**A**) pig density and (**B**) human population density. Areas with below-average pig or human density for the region (≤40 pigs/km^2^ or ≤275 people/km^2^) are not visualized.

**Fig 4:**
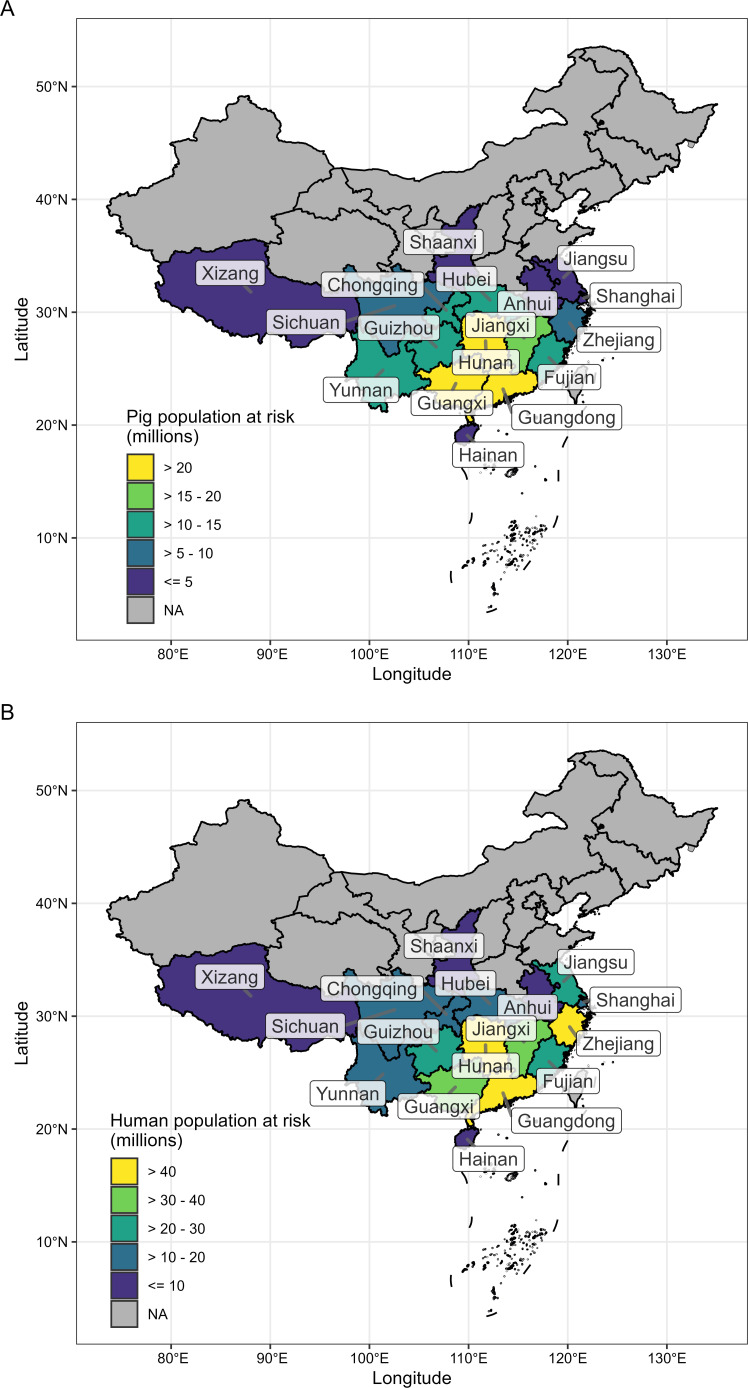
Total (**A**) pig and (**B**) human population at risk of SADSr-CoV spillover by Chinese province. “At risk” here is defined by the number of pigs or people within an area predicted as suitable for at least one rhinacovirus bat host.

The largest regions of above-average pig density (>40 pigs/km^2^) within suitable habitat for rhinacovirus host species were found primarily in southern China, throughout Vietnam, and parts of Myanmar, Thailand, and eastern India (Fig. 3A). The areas with the highest pig density (>500 pigs/km^2^) occurred mostly in China, where Hunan, Guangxi, and Guangdong provinces contained the highest numbers of pigs (>20 million) overlapping with suitable host habitat (Fig. 4A), as well as northern Vietnam and central Thailand. Regions with above-average human density (>275 people/km^2^) within suitable host habitat were observed mainly in southern China, Java, and eastern India (Fig. 3B). Regions with the highest human density (>2,000 people/km^2^) could be identified near cities such as Jakarta (Indonesia), Bangkok (Thailand), Hanoi (Vietnam), and Shenzhen (China). Within China, Guangdong, Hunan, and Zhejiang provinces contained the highest number of people (>40 million) living within areas of suitable host habitat (Fig. 4B).

## DISCUSSION

Our analysis provides novel insights into the diversity, evolutionary origin, and spillover potential of the rhinacoviruses, a sub-genus of alphacoronaviruses that includes SADS-CoV, a recently emerged enteropathogenic virus affecting swine (28). SADS-CoV is also able to infect primary human airway epithelial cells *in vitro* (33) and cause lethal disease in suckling mice (36), therefore representing potential for zoonotic spillover or infection of other mammalian taxa. As with several other CoVs that infect pigs and other livestock (88), SADS-CoV appears to have originated from bats—with previously reported SADSr-CoV sequences mainly from *Rhinolophus* spp. bats in Asia (20, 26).

Our phylogenetic findings are consistent with those of previous studies based on full genomes but expand the known host and geographic range of bat rhinacoviruses in Southeast Asia and reveal a more complex viral diversity (20, 25, 26). Although the lack of reliable data on overall sample size for most of the GenBank data (i.e., lack of negative data) meant it was not possible to estimate prevalence, the large geographic range of rhinacovirus hosts, their diversity, and the known cross-species potential of SADSr-CoVs suggest that efforts to better assess spillover risk of this CoV subgenus are valuable. Our paper analyzes an additional 186 new *Rhinacovirus* RdRp sequences from China to reveal a large, previously underappreciated diversity of rhinacoviruses circulating in 15 bat species from four genera in 12 Chinese provinces. This data set encompasses four additional Chinese provinces and eight additional host species compared to previous rhinacovirus phylogenies available in the literature (25). Similarly to the full genome phylogeny of Hassanin et al. (25), our phylogenetic analyses reveal the presence of two distinct monophyletic clades with an average of 83.6% nucleotide identity between them (across a short fragment of the RdRp gene) (Fig. 1 and 2). This value is well below the 90% threshold proposed for CoV species demarcation by (89) using this same region of the RdRp gene. Clade 1 viruses were predominantly associated with *R. pusillus* but, contrary to Hassanin et al. (25), which detected Clade 1 exclusively in *R. pusillus*, our data suggested that Clade 1 has also been detected in other *Rhinolophus* species and other bat genera (*Myotis*, *Hipposideros,* and *Tylonycteris*) (Fig. 1). The detection of rhinacoviruses in non-*Rhinolophus* host species was limited (*n* = 1 for each species, Table 1), and likely represents rare cross-species transmission events in natural bat communities, although we cannot rule out species misidentification. Based on our RdRp phylogeny, *R. pusillus* has been identified as the most likely ancestral host (i.e., the host of the progenitor viruses from which the clade evolved) of Clade 1 (Fig. 1). However, we could not confidently identify the geographic location of origin for Clade 1 viruses (Fig. 2).

Our RdRp phylogeny identified *R. sinicus/thomasi* as the most likely ancestral hosts, and Yunnan as the most likely evolutionary origin of viruses in Clade 2 that are associated with SADS-CoV (Fig. 1 and 2). Our extensive data set revealed a more complex phylogeographic structure within Clade 2 than previously observed using whole-genome data (25). Five well-supported lineages mostly evolving in distinct bat assemblages (*R. sinicus/thomasi*, *R. affinis,* and *R. stheno*) and circulating in Yunnan were retrieved in our phylogenetic trees (Fig. 1 and 2). The same Hainan lineage as in Hassanin et al. (25) was identified in *R. affinis,* but it included a sequence from Guangdong province, suggesting some dispersal between Hainan and mainland China and contradicting the hypothesis of allopatric evolution on Hainan Island suggested by Hassanin et al. (25). We also identified a rhinacovirus RdRp lineage distributed in southeastern China (Fujian, Zhejiang, Anhui, Jiangxi, Hubei, Hunan, Guangdong, and Guangxi) and mostly evolving in *R. sinicus/thomasi* and *R. affinis* that was not identified in previous phylogenies of rhinacoviruses (25). Our ancestral host reconstructions based on the RdRp gene repeatedly found that *R. sinicus/thomasi* was the most likely ancestral host of the diverse assemblage of rhinacoviruses in Clade 2, suggesting that this species has played a major role in the evolution and spread of SADSr-CoVs and, with *R. affinis*, represents important reservoirs of rhinacovirus diversity in the region (Fig. 1). However, bats in countries adjacent to the southern border of Yunnan (Laos, Myanmar, and Vietnam) appear to be highly undersampled for CoVs (8), and further viral discovery in wildlife in these countries may yield further information of relevance to SADSr-CoV evolution (25). This is further supported by the diversity of other alpha-CoVs recently reported from a range of sites across China (90–94).

Within Clade 2, using all available data from our field surveillance and published sequences on GenBank, our RdRp analyses identified *R. affinis* as the most likely origin host for SADS-CoV emergence in pigs (i.e., the host from which the initial spillover event occurred) (Fig. 1). This result is robust, in that it was identical in all our barcoded and randomly subsampled data sets. Similar results were also obtained when using a smaller data set of rhinacovirus whole genomes (25). Cross-species transmission of rhinacoviruses between bat species appears to be a regular feature of these viruses, as has already been observed for bat CoVs in general (8), and may be driving the spread of rhinacoviruses into multiple lineages and species. The index ratios we reported for both location and host suggest that transmission of rhinacoviruses between hosts and locations in southern China is infrequent (Table 3). However, the substantial number of recombination events reported using whole-genome analyses of bat-CoVs suggests that this may represent an underestimate of cross-species gene flow (25, 95, 96). Further research on the ecological, phylogenetic, and behavioral factors that determine interspecies transmission among bats is warranted, along with increased sampling and full genome sequencing. Characterizing additional rhinoviruses from yet-to-be-sampled bat or other wildlife species may change our understanding of ancestral hosts and other phylogeographic patterns.

SADS-CoV sequences from pigs in both Guangdong and Fujian provinces obtained in 2017 and 2019 formed a single clade in our phylogenetic trees (Fig. 2). The sequence from the most recent outbreak in Henan in 2023 also belonged to the same clade (32). Most of these sequences, including the single Fujian sequence from 2019 (29), were identical in the RdRp gene fragment used in this study, and the remaining two sequences varied by only a single-nucleotide substitution each. This is consistent with a recent origin for SADS-CoV (26, 28) and suggests that SADS-CoV arose from a single initial spillover event, most likely from *R. affinis*, as previously proposed in other studies using smaller data sets of full genomes (25, 26, 32). Consistent with previous studies (20, 21, 26, 28, 30, 35), we also identified Guangdong province as the most likely geographic origin of SADS-CoV (Fig. 2), with the proviso that sampling of bats and pigs in neighboring countries has been sparse. This is particularly important given that the clinical signs of SADS in pigs are very similar to other diarrheal diseases, so that the SADS-CoV may have been cryptically involved in multiple outbreaks (20). It is also important to note that SADS-CoV has usually been reported along with other CoVs that cause diarrheal disease, including in the 2021 Guangxi report (31), suggesting that past outbreaks that identified one viral etiology may have missed the presence of SADS-CoV.

An important limitation of this study is that only partial RdRp sequences were generated and used in our phylogenetic analysis. Even if the topology of our RdRp phylogeny is largely consistent with whole-genome phylogenies (25, 26, 32), it is important to note that using more complete genomes might provide different ancestral reconstructions. However, as whole-genome sequences are available for only 12 of the 481 bat samples from which a RdRp sequence has been analyzed in this study (Fig. 2), our RdRp phylogeny still brings important insights into the evolutionary history of rhinacoviruses that could not be obtained otherwise. The Spike protein genes of SADS-CoV and HKU2-CoV display a different evolutionary history than the rest of the genome and are more closely related to rodent CoV (26), which likely results from a recombination event with a betacoronavirus (23, 27, 28). However, our S gene phylogeny also supports an *R. affinis* origin for SADS-CoV. These recombination events remain undetectable when using only RdRp sequences. These short RdRp sequences also limited our ability to reconstruct the spatiotemporal dynamics of rhinacoviruses and SADSr-CoVs due to the lack of temporal signal in our data set. Full genomes would enable a more rigorous assessment of the phylodynamics of these viruses. However, it should be noted that the lack of temporal signal was also observed in a limited data set of *Rhinacovirus* and SADSr-CoV full genomes (26), which might be explained by the short timeframe (3 years) during which these genomes were obtained.

The transmission pathway of SADSr-CoVs from bats to pigs remains poorly understood. Some authors have suggested that rodents may have been involved in SADS-CoV transmission between bats and pigs (26, 28), as it has been demonstrated that SADS-CoV can infect rodent cell lines (35), and rats were frequently observed in pig facilities (28). This is a plausible transmission pathway as CoV screening in Vietnam has shown that several rodent species tested in local wildlife farms were infected by bat CoVs closely related to PEDV, another alphacoronavirus infecting pig populations (97). However, during our field investigations at two of the farms where SADS-CoV was identified in Guangdong province, small insectivorous bats were observed roosting under the roof of several pig houses, and bat feces were found around some pig houses (unpubl. obs.). SADSr-CoVs have been identified commonly from bat feces or fecal smears, and like most other known bat-CoVs, are likely to predominantly infect the intestines of bats, even though they may be detected in other organs (98, 99). These findings suggest fecal contamination of pig food or fomites, or direct bat-pig contact in pig farms, is a likely route of transmission of SADSr-CoVs from bats to pigs, and that the presence of an intermediate host may not be necessary for spillover. Contact between pigs and bats has played an important role in the transmission of other bat viruses to pigs and humans, including Nipah virus in Malaysia (100) and Menangle virus in Australia (101). Future ecological investigations to understand bat behavior and bat-pig interactions at these sites, including the extent and seasonality of roosting, foraging, and other activity patterns, as well as the temporality of viral shedding, would likely be valuable for spillover prevention.

Despite the strong associations of some *Rhinacovirus* clades and subclades with a specific *Rhinolophus* species, there was slightly stronger clustering by location than by host across the complete data set (Table 3). Sequences sampled from Yunnan, Guangdong, Guangxi, and Hainan provinces formed large, well-supported monophyletic subclades and were characterized by high negative values of SES MPD, suggesting that there may be independent evolutionary lineages circulating in these locations (Table 2). This evidence of limited long-distance transmission of rhinacoviruses in the region may be related to the ecology of their *Rhinolophus* host species. *R. affinis*, *R. sinicus/thomasi*, *R. pusillus*, and *R. stheno* are most commonly found in disturbed and undisturbed forests of Asia, where they roost in caves and buildings (102–106). *Rhinolophus* flight ability is adapted to the forest interior, where they forage for insects in the understory, and these species rarely fly long distances in open habitats (107), and wing morphology is a strong predictor of population genetic structure in bats (108). The sharing of specific virus lineages among *Rhinolophus* species is likely facilitated by their frequent co-roosting and overlap in foraging sites (109).

Although substantial bat sampling was conducted in central northern and northern China for this study, most rhinacoviruses were discovered in central, southern, and southwestern China, and most subclades within clade 2 were in samples from southern (Guangdong and Hainan) and southwestern (Yunnan) China. These regions are recognized hotspots of CoV phylogenetic diversity and also harbor a substantial diversity of sarbecoviruses (6, 110, 111). This study further supports the important role of these regions in CoV diversification and emergence in Asia (8). However, rhinacoviruses have also been detected in Thailand and Vietnam, suggesting that the circulation of these viruses is not limited to China, and as with SARSr-CoVs (112, 113), likely spans much of Southeast Asia. Increased bat sampling and CoV screening in neighboring countries such as Vietnam, Laos, Myanmar, Thailand, and Cambodia may provide a better understanding of the distribution and evolution of these viruses.

Our species distribution modeling showed that southern China and Vietnam contain the largest regions of suitable habitat for three reservoir hosts of rhinacoviruses (*R. affinis*, *R. pusillus*, and *R. sinicus/thomasi*) and overlap with areas of high pig and human population densities (Fig. 3 and 4). Other countries also had localized regions of overlap between reservoir and recipient hosts, such as Indonesia (specifically Java), Myanmar, Thailand, and eastern India (Fig. 3 and 4). We note that geographic co-occurrence of reservoir and recipient hosts is just one component in the process of spillover, and therefore, the areas highlighted in our maps are not necessarily sites with the greatest spillover risk. Other data (e.g., CoV host prevalence, reservoir host abundance; seasonality in viral shedding; the frequency and types of contact between bats, farmed animals, and people; recipient host immune defenses) are needed to build a fuller picture of where and how spillover may be occurring (52). Future studies to develop and use rhinacovirus-specific serological assays for surveillance in bats, livestock, and people could lead to additional insights into a natural host range and cross-species transmission. Within China, Hunan and Guangdong were identified as provinces where the largest pig and human populations live within habitat suitable for rhinacovirus bat hosts (Fig. 3 and 4). In addition, the presence of large pig farms and dense, interconnected villages, towns, and cities in these regions suggests that future SADS-CoV spillover events in pig farms in these regions may have considerable consequences. Considering the potential for SADS-CoV to infect humans (33), this may also put large human populations at risk of direct (via bat reservoirs) or indirect (via an intermediate host) infection. The clinical signs of SADS in pig populations closely resemble other predominantly piglet diarrheal diseases, including PEDV, which was also present in the same pig herds involved in the first-reported SADS outbreak (20). Thus, it is plausible that cryptic outbreaks of SADS occur, and human exposure to large quantities of virus in pig feces would be likely, particularly through management of a diarrheal disease outbreak, and given the practice of passive immunization of pig herds (feeding of intestines from dead piglets) by farmers occurs widely (114–116). Increased pig and human surveillance in or near swine farms in these regions is therefore warranted to detect bat CoV spillover events before they could cause large outbreaks and may help limit their impact. This would likely be most effective if targeted to people with occupational risk of exposure (e.g., pig farmers) and those living close to *Rhinolophus* spp. bat roosts and foraging sites. Even if human infection by SADS-CoV is benign, it would likely result in seroconversion, suggesting serological surveillance would be effective in screening people. While no human cases of SADS-CoV infection have been identified to date, the reported number of people tested by PCR or serology is low (<50) (20) compared to the population of tens of millions within this region, and the high potential for occupational exposure.

## Data Availability

All RdRp sequences generated for this study are available in GenBank with accession numbers OK392027–OK392038, OK392040–OK392048, OK430982–OK431065, OK431067–OK431078, OK480528, OK480530–OK480555, and OK480557–OK480598. Sequence metadata are available in Table S1. The code used for bat host distribution modeling can be found at https://github.com/ecohealthalliance/SADSr-CoVs and Zenodo https://doi.org/10.5281/zenodo.14188369. Supplemental material is available at https://osf.io/rcepg/?view_only=f673c894413a44df83716db155aad489 and https://naturehealthglobal.org/latinne-et-al-2025-supplemental/).
